# Health, Work, Invisibilities and Collective Resistance in an Asbestos-Exposed Territory in the Pedro Leopoldo Region, (MG), Brazil

**DOI:** 10.3390/ijerph23030315

**Published:** 2026-03-04

**Authors:** Eliana Guimaraes Felix, Alexandro Cristino Guimaraes

**Affiliations:** 1 Center for Studies on Worker Health and Human Ecology (CESTEH), National School of Public Health (ENSP), Oswaldo Cruz Foundation (Fiocruz), Rio de Janeiro 21041210, RJ, Brazil; 2 Brazilian Association of Asbestos-Exposed Workers of the State of Minas Gerais, Pedro Leopoldo 33251008, MG, Brazil; alexandrocristinog9@gmail.com

**Keywords:** asbestos, health, work, occupational health, environmental injustice

## Abstract

**Highlights:**

**Public Health Relevance—How does this work relate to a public health issue?**

- This study reveals a silent, persistent, and structurally invisibilized epidemic of asbestos-related diseases in Pedro Leopoldo (MG), Brazil, marked by decades of environmental injustice, state omission, and institutional underreporting, particularly among historically vulnerable populations.
- It demonstrates that occupational, domestic, and environmental exposures continue to affect the community even after the national asbestos ban, exposing a neglected sanitary and environmental liability and reinforcing the urgency of protective public health policies and surveillance actions.

**Public Health Significance—Why is this work significant to public health?**

- The findings highlight gender inequities, intergenerational contamination risks, and profound diagnostic invisibility, providing unprecedented evidence on the health of asbestos-exposed Brazilian populations, such as those in Pedro Leopoldo/MG.
- The research offers robust qualitative evidence to strengthen health surveillance, improve the recognition of asbestos-related diseases, and guide equitable public policies in territories historically marked by intensive use and consumption of the mineral.

**Public Health Implications—What are the key implications for practitioners, policymakers and/or researchers?**

- The study underscores the need for integrated epidemiological surveillance, historical reparation policies, and the development of tailored care pathways for asbestos-exposed populations within the Brazilian Unified Health System (SUS).
- It highlights the central role of exposed workers and their collective organization as a movement of resistance, particularly through the Brazilian Association of Asbestos-Exposed Workers of Minas Gerais (ABREA/MG), in confronting necropolitical invisibility, expanding access to care, and contributing to more just, territorially grounded, and community-responsive public health actions.

**Abstract:**

Asbestos, a group 1 carcinogen, has generated a serious health and environmental liability in Pedro Leopoldo/MG, Brazil, even after its national ban in 2017. This study aims to analyze the silent epidemic of asbestos-related diseases (ARDs) through the lens of social injustice. We used a qualitative, socio-historical, and clinical approach within the framework of an Expanded Research Community (ERC), based on ergology, with content analysis of interviews with workers and institutional documents. The evidence reveals a pattern of institutional silencing and omission, marked by corporate fraud, denial of risk, and medical underreporting, perpetuating occupational, domestic, and environmental exposure. In response, the Brazilian Association of Asbestos-Exposed Individuals of Minas Gerais (ABREA/MG) emerged as a central actor in the struggle for recognition and justice. It is concluded that overcoming this injustice requires structured public policies of recognition, integrated surveillance, historical reparation, and strengthening of the SUS (Unified Health System), with collective resistance being fundamental to transforming suffering into memory and social demands.

## 1. Introduction

Asbestos is recognized by the World Health Organization (WHO) [1] as one of the most widespread occupational carcinogens, being associated with a large proportion of work-related cancer cases [2,3]. The International Agency for Research on Cancer (IARC) [4] classifies asbestos as a Group 1 substance, that is, proven to be carcinogenic to humans. Despite the ban on its use in Brazil since 2017, asbestos remains a serious public health problem, due to the accumulated health and environmental liabilities over decades of exposure [5,6,7].

Brazilian asbestos exposure standards have always proven inadequate when compared to international standards [8]. The national tolerance limits, defended by business sectors, were set at values up to twenty times higher than those allowed in the United States, even though epidemiological studies had already proven the non-existence of safe exposure levels. Regulatory Standard No. 15 (NR-15), of the Ministry of Labor [9], for example, established a limit of 2.0 fibers/cm^3^ for respirable chrysotile asbestos fibers. This parameter was considered unsafe and arbitrary, compromising the health of workers. Social and labor movements that worked to defend the definitive ban on asbestos in Brazil denounced these issues.

In fact, even after the domestic ban, Brazil remained the world’s third-largest exporter of asbestos, perpetuating a global chain of exposure and contamination. In 2023, approximately 190,000 tons of the fiber were exported, mainly to India, Indonesia, and Bangladesh [6,7,9,10,11]. This scenario amplifies the transnational impacts on health and the environment, contradicting the national ban.

Asbestos-Related Diseases (ARD) have long latency periods, which can reach four decades [2,3,12,13,14,15,16,17,18,19,20,21,22]. Even after exposure is interrupted, the health effects persist, keeping the population at high risk. In this context, malignant mesothelioma—a rare and highly lethal neoplasm, whose etiology is associated with asbestos in up to 90% of cases—is recognized as a sentinel disease [23,24,25], its incidence being an indicator for surveillance and identification of regions that have suffered exposure to the fiber.

Given this scenario, projections for mesothelioma mortality in Brazil suggest a peak within the 2021–2026 window, within forecasts extending to 2030 [26]. The seriousness of the local situation is evident: between 2000 and 2017, the municipality of Pedro Leopoldo (MG) presented mortality rates from lung and ovarian cancer higher than those of more populous municipalities that also used asbestos [27]. This data is a strong indication of the severity of local exposure, classifying the region as having high consumption and use of the fiber.

The municipality of Pedro Leopoldo (MG), with an estimated population of 65,174 inhabitants for 2025 [28], and neighboring localities (Matozinhos, Confins, São José da Lapa and Vespasiano) concentrated, for more than five decades (1973–2019), industrial activities based on the processing of chrysotile asbestos and, possibly, amphiboles. Both types are proven carcinogenic agents. This activity directly and indirectly exposed thousands of people to the risk of illness, resulting in occupational, domestic and environmental exposures. Over these decades, asbestos processing became structurally embedded in the local economy and shaped work trajectories and daily life in the municipality and its surroundings [21,22].

This article aims to analyze the ways in which illness is made invisible, the dynamics of (in)security and (lack of) protection at work, collective resistance and the social and institutional repercussions of confronting those exposed to asbestos liabilities in Pedro Leopoldo (MG), based on a qualitative, participatory and interdisciplinary approach. The aim, therefore, was to understand the relationships between work, environment, health, and resistance in contexts marked by prolonged exposure to asbestos and institutional silencing.

In analytical terms, regarding theoretical frameworks, this research is based on the lenses of environmental justice, which interpret how responsibilities related to asbestos are unequally distributed and politically managed over time, guiding this study. These frameworks help to situate the invisibility of the disease, (in)security at work, and institutional silencing within broader power relations that shape which lives are protected, which exposures are normalized, and which harms become socially and administratively legible.

In this sense, this study addresses both the health and environmental liabilities resulting from occupational and household exposure to asbestos, as well as the health problems resulting from past exposures. Considering the long latency periods and the irreversible nature of asbestos-related diseases, the current burden of illness reflects historical exposure processes, while also highlighting ongoing risks associated with environmental contamination and the inadequacy of remediation actions in response to the harm and damage suffered.

## 2. Materials and Methods

This is a qualitative, exploratory and participatory research, based on the frameworks of Occupational Health and Latin American Collective Health [29,30,31,32], in constant dialog with the principles of Environmental Justice [33,34]. The study articulated methods of social sciences in health and action research through the formation of an Expanded Research Community (ERC) [35,36], whose objective was to recognize the research subjects as protagonists in the production of knowledge about their own territories and experiences of illness.

From an ethical point of view, the research was conducted in strict compliance with the rules of Resolution No. 466/2012 and 510/2016 of the National Health Council, which regulate ethics in research involving human beings in Brazil. The study ensured respect for the dignity, autonomy, and confidentiality of the participants and was approved by the Ethics Committee of ENSP/FIOCRUZ (CAAE 03323018.4.0000.5240). All subjects were duly informed about the objectives, risks, and benefits of the study, expressing their free and informed consent, in accordance with the precepts of bioethics [37].

The participation of the Brazilian Association of Asbestos-Exposed Individuals of Minas Gerais (ABREA/MG) was central to ensuring the transparency of the process, the social feedback of the results, and the collective construction of interpretations. This partnership reinforced the ethical-political commitment inherent in action research in occupational health, articulating scientific production and social justice [29,30,35,36].

The research followed the frameworks of ergology, which recognizes workers as co-producers of knowledge and values the dialog between scientific knowledge and experiential knowledge [36]. The research was conducted by a researcher native to the studied region, with experience in the field of occupational health and environmental justice. This territorial insertion facilitated access to participants and the building of trusting relationships, especially in a context marked by institutional silence and lack of assistance. Recognizing the potential effects of this proximity on the analytical process, reflexive practices were adopted, including field notes and collective discussions within the ERC, with the aim of expanding, sharing, and validating guidelines, knowledge, actions, and interpretations of the social actors participating in the research, including workers, activists, and researchers.

Recruitment was guided by criteria of gender balance and diversity of social roles, including directly exposed workers and family members with a history of indirect exposure. The company no longer employed these workers at the time of the research. The researcher interviewed all participants, with the support of the ABREA/MG, whose social legitimacy was fundamental in locating people residing approximately the company. This search covered the municipality where the industry was headquartered (Pedro Leopoldo) and neighboring municipalities such as Confins, São José da Lapa, Vespasiano, and Matozinhos. These municipalities also supplied labor to the industrial plant.

Data collection took place between July 2021 and October 2022. Although the period coincided with the COVID-19 pandemic, all fieldwork stages strictly followed health protocols, ensuring safe conditions for researchers and participants. The study involved 36 participants, men and women in balanced proportions, through in-depth interviews and a group meeting.

The interviews, lasting up to two hours, addressed work trajectories, risk perceptions, experiences of illness, and forms of resistance and social mobilization. This process allowed access to the subjective and material dimensions of the participants’ experiences and was directly linked to the construction of the ERC, formed by researchers, workers, and local activists. In this context, the CAP acted as a methodological component, integrating narratives, observations, and collective analyses of the symbolic, political, and territorial dimensions of environmental contamination [38].

The meeting and interview records were organized and analyzed using the content analysis technique [39]. The verbal and nonverbal material went through stages of pre-analysis, exploration, categorization, and interpretation, resulting in the construction of analytical categories based on similarity, frequency, and relevance to the research objectives. The systematization and organization of the categories were supported by the MAXQDA^®^ software Analytics Pro 22 6.0 [40], a tool used for handling unstructured qualitative data, allowing integrated analyses, rigorous categorization, and multiple ways of visualizing the results [41,42].

The research adopted a participatory and dialogical approach. The preliminary results were presented in open meetings for discussion and collective validation, allowing participants to reinterpret and actively contribute to the refinement of the analyses [35,36,43,44]. The shared construction of knowledge ensured methodological rigor, social legitimacy, and a scientific practice guided by environmental justice and the recognition of the subjects involved.

## 3. Results

**Figure 1:**
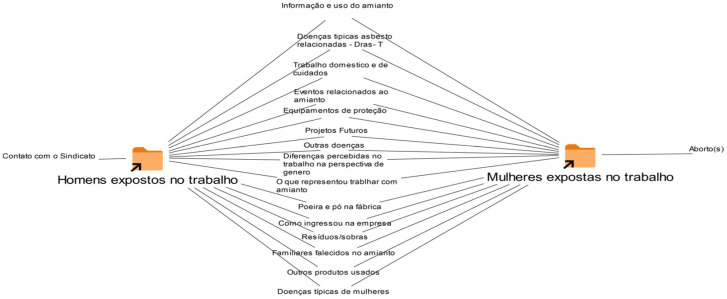
Comparative organizational chart demonstrating asymmetries and convergences in occupational exposure to asbestos by gender. Comparative diagram of the main categories extracted from interviews with asbestos-exposed individuals in the studied region (two-case model). The figure compares and articulates the narratives around: Informação e uso do amianto (Information and use of asbestos); Doenças típicas asbesto-relacionadas—DRA-T (Typical asbestos-related diseases—ADR-T); Trabalho doméstico e de cuidados (Domestic and care work); Eventos relacionados ao amianto (Asbestos-related events); Equipamentos de proteção individual (Personal protective equipment); Projetos futuros (Future projects); Outras doenças (Other diseases); Diferenças percebidas no trabalho na perspectiva de gênero (Perceived work differences from a gender perspective); O que representou trabalhar com o amianto (What working with asbestos represented); Poeira e pó na fábrica (Dust and powder in the factory); Como ingressou na empresa (How they entered the company); Resíduos/sobras (Residues/waste); Familiares falecidos no amianto (Family members deceased due to asbestos); Outros produtos usados (Other products used); Doenças típicas de mulheres (Typical diseases among women); Homens expostos ao amianto (Men exposed to asbestos); Contato com o sindicato (Contact with the labor union); Mulheres expostas ao amianto (Women exposed to asbestos); e Aborto(s) (Miscarriage[s]).

The comparative organizational chart presented in Figure 1, developed from the categories coded in the MAXQDA^®^ software [40], synthesizes the similarities and differences identified in interviews conducted with men and women occupationally exposed to asbestos. This analytical tool made it possible to systematize, in an integrated way, the points of convergence and the distinctive aspects between the groups, allowing a precise visualization of the dynamics of exposure, working conditions and the differentiated effects on health.

The graphic representation was organized to visually highlight the degrees of convergence and divergence between the groups. Categories common to both men and women were positioned in the central area of the diagram, indicating shared experiences of exposure, working conditions, and health effects. As the categories showed greater specificity or differentiation between the groups, they were progressively scaled towards the edges of the figure. In this way, the most divergent points—those mentioned exclusively or predominantly by one of the groups—are located at the lateral extremities of the diagram, allowing for a gradual and comparative reading of the identified asymmetries.

The analysis reveals that most of the categories related to work dynamics and health effects are concentrated in the area of intersection between the groups. This indicates that men and women share a significant set of experiences of risk, vulnerability, and illness. The distinctions, in turn, are predominantly located at the base of the model, with emphasis on aspects such as the insufficiency and inadequacy of Personal Protective Equipment (PPE), the asymmetry in access to information on the use of asbestos, the management of industrial waste, and continuous exposure to dust and powder generated in production processes.

In line with evidence presented in other sections of this study, there are also reports of family members who died because of ADR, as well as references to other chronic diseases and conditions that occur more frequently in women. This set reinforces the persistence of epidemiological invisibility and the institutional gap in health care for this population.

The political-organizational dimension presents a relevant asymmetry: only the participants in the male group reported mobilization and resistance processes, initiated from the approach to the local union. Among the women, no mentions of forms of collective articulation or references to the social movement were identified, which may indicate structural barriers to female participation in these spaces or the invisibility of their organizational trajectories.

Finally, a highly sensitive category emerges exclusively in the female group: several accounts mention episodes of abortions throughout their working lives. This empirical data, given its complexity and implications for the debate on reproductive health in contexts of environmental and occupational exposure to toxic agents, signals the need for specific investigation and analytical deepening in subsequent studies.

### 3.1. Sociodemographic Conditions of the Participants

The results of this study highlight the complexity of the scenario experienced by workers exposed to asbestos in the Pedro Leopoldo region, articulating sociodemographic, labor, environmental, and gender dimensions. These dimensions intertwine in addressing the health, generational, and environmental liabilities caused by asbestos exposure. The sample totaled 36 participants, distributed evenly between 18 men and 18 women, all of whom had already been formally dismissed from the company at the time of the interviews.

The socio-demographic conditions presented by the participants consisted of a predominant age range for both sexes, situated between 60 and 70 years. Regarding education, primary education prevailed, with illiterate men identified, but no women in this condition. Only women reported higher education. The majority were retired, with a higher proportion of women, and retirement, mainly due to length of service, was the main source of income. In terms of race/color, women predominantly self-identified as Black, and men as mixed-race. The population was distributed in a balanced way, with 18 men and 18 women. All men reported a history of work with occupational exposure, while women presented, proportionally, links between occupational and non-occupational exposure in domestic and family contexts. The majority resided in Pedro Leopoldo, despite the territorial scope studied involving other surrounding municipalities.

Regarding education, primary education prevailed, and it is relevant to note the presence of men with illiteracy, but no women in this situation. In contrast, higher education was identified exclusively among female participants. Most participants were retired, with a higher proportion of women, with retirement due to length of service being the main source of income.

### 3.2. Multiple Exposures, Gender Inequalities, and Permanent Environmental Contamination

This study revealed a scenario of multiple and persistent exposures, permeated by social and gender inequalities, institutional negligence, and the absence of environmental and health surveillance policies. The case of Pedro Leopoldo (MG) constitutes a paradigmatic example of the historical invisibility of asbestos-related diseases and state omission in the face of risks widely documented in the scientific literature [45,46,47,48,49,50].

Working conditions were marked by high dust concentration, absence of exhaust fans, poor ventilation, and lack of physical barriers. The participants’ testimonies attest to the daily violence and the naturalization of precariousness:

[…It was an exhausting routine, every day, at the cullet mill. The [asbestos] packages would arrive, some already burst, and we had to take what was left and transfer it to another bag. It was hard work, throwing everything with a shovel into a new bag, sweating and getting more and more tired…]

(H15)

[…I had to clean a large tank full of water and chemicals. When I left there, I couldn’t touch anything with my hand or leg, because I would get injured and bleed because muriatic acid, cement, asbestos and other mixtures were used. Sometimes, the wounds would turn white, like pork without skin…]

(H15)

The use of aggressive chemical agents, such as muriatic acid, in material recovery tanks, and the direct handling of asbestos were common routines. The workers dealt with dust that “imitated smoke” and remained in it while repairs were carried out:

[…It’s a shard mill…Then they would throw that smoke, which was dust… People would go into that mill to make adjustments to fix it and come out all white…]

(H15)

The literature already showed that, even when workers used the officially offered personal protective equipment, such as masks and gloves, these devices were not able to guarantee real barriers against exposure to asbestos [51]. The scarcity and limitation of PPE models forced many to resort to improvised solutions, keeping the risks largely uncontrolled. The account of one participant illustrates the continuous exposure:

[…I only wore a rubber boot and the clothes we wore were the ones we brought from home. When we finished our shift, we went home in the same clothes. At the time, I didn’t have hair on my arms and stomach, because the sores were frequent. All this without mentioning the hearing problems that arose after years of working there. We used gauze to plug our ears…]

(H15)

Another account from the same participant demonstrates the uselessness of the protection offered:

[…The mask that was offered was almost useless. It could only be changed three times a week, even if it was wet and black with sweat…]

(H15)

These testimonies illustrate the daily violence of precarious work and the absence of effective worker health policies. Physical and psychological suffering is dramatically manifested in the account:

[…During dinner time, when I lay inside the big bags, full of asbestos, I asked God to take my life, because I couldn’t take it anymore.]

Diagnosed with asbestosis, he questions the medical and company conduct:

[Periodic exams carried out by the factory always considered him “fit,” until an external campaign, promoted by health institutions, detected the disease…]

Despite the serious and proven link between asbestos exposure and illness, the company used fear-mongering (threats of unemployment and factory closure) to dissuade workers, the community, and municipal authorities from taking actions that could negatively affect production. Unemployment for those who worked with chrysotile asbestos was a constant and unfounded threat, maintained while asbestos was in use:

[…they are saying many times we are thinking about closing but we don’t want to close the factory we want the factory to stay right […] because if the factory closes how will there be profit? it would be better if the factory doesn’t go bankrupt and everyone has work…to earn their bread]

(M5)

The study also recalled a strike episode in 1986, in which the company hired women from the community on an emergency basis to replace striking workers, ignoring the legal prohibition of women’s activities in unhealthy environments [23,52]. These women were subjected to degrading conditions, without access to toilets and PPE, being used as an instrument to weaken worker mobilization.

[…then we went on strike. Then a lot of women were hired. Then the women covered for the striking workforce…]

(H15)

The risk is amplified by the inadequacy of PPE to the female body. Greenberg and Dement [53] highlight that the absence of equipment such as respirators and gloves designed for women increases vulnerability to exposure, corroborated by research in Korea [54]. A local account demonstrates the physical consequence of this inadequacy:

[…The glove didn’t fit, it was too big. So the calender [machine] swallowed my hand. I broke it and it’s been crooked ever since…]

(M21)

Gender inequality permeated the entire production process. In addition to receiving lower wages and being exposed to moral and symbolic discrimination, women had their structural vulnerability accentuated by the sexual division of labor in a masculinized factory environment [55,56,57,58].

[…Salary, women always earn less […] I had male colleagues who earned more, even doing the same job…]

(M22)

[There was only a bathroom for men. I went without showering and without going to the bathroom, when I needed to go I went to the office]

(M12)

The problem of asbestos is not limited to exposed workers, but affects the whole of society, including family members, neighbors, and non-occupationally exposed and unmonitored populations [12,46]. This compromises the health of future generations, aggravated by the loss of exposure history and memories.

The handling of toxic substances has direct effects on reproductive health, potentially compromising fertility, causing genetic mutations, and affecting pregnancy, according to the International Labour Organization [59]. There is the risk of passive transmission of fibers through work clothes, skin, and hair, affecting family members and breastfed babies [60]. This intergenerational dimension of contamination requires active surveillance and public policies for full reparation.

Corroborating the literature, the findings of this study regarding abortions were frequent in the female group, drawing attention to their incidence. A total of 12 (twelve) abortions were reported, many of which were recurrent, occurring during the work period in a hazardous environment and with the use of carcinogenic substances, corresponding to approximately 70% of the women interviewed.

[I had 3 miscarriages]

(M2)

[…Then I had a miscarriage, I was off work… Then the doctor spoke to me, -it’s either the service or you… I said, -no, I’m married, I have a child, that’s not all I lost. Then he said, -then in that case there’s no way, they won’t accept this certificate. So I accepted it, if it doesn’t work, it doesn’t work. Then I never went back…]

(M28)

[I’ve had miscarriages–two]

(M1)

Although recurrent miscarriage may be related to multiple factors (hormonal, anatomical, genetic), the temporal correlation and the context of exposure to carcinogenic and toxic agents suggest the need to investigate the etiology of these cases to trace the epidemiology and causal link of the harm resulting from asbestos exposure [60]. Possibly, this is yet another generational liability with a significant impact on the exposed region.

### 3.3. The Geography of Invisible Contamination and Water Injustice

The participants’ accounts demonstrate a geography of invisible contamination, in which industrial waste was irregularly disposed of in open areas, close to watercourses and the river that supplies the city, aggravating water and soil contamination [21].

A crucial testimony details the illegal management of waste, including the most dangerous types of asbestos:

[…At the time they used blue asbestos… But the blue one was banned because it was more dangerous. The blue and brown ones that were in stock there, they buried everything in the landfill, near the river, at the factory itself. It was all buried there. Several batches of asbestos, even today, near the river, and there is a spring that comes up there on the BR, it is channeled and passes through the factory and passes near the river where this stock is. And there is also the water table which has two artesian wells. Near the artesian well there, everything is covered in asbestos…]

(H34)

The illegal disposal of blue and brown asbestos (amphiboles) near the water table, springs, and artesian wells is an extremely serious empirical finding for the discussion of environmental injustice. The hierarchical organization of the factory space overtly reproduced class inequalities, characterizing water injustice: while engineers and supervisors occupied ventilated sectors and consumed mineral water or water from exclusive artesian wells, the workers were forced to use water from industrial waste disposal areas.

[…The water you bathed in there was like bathing in the stream… the water didn’t come out clear. It came out yellow. The water there was undrinkable. They [Administrative staff] drank mineral water and we drank that garbage there.] [Mineral only they take…]

(H19)

This inequality therefore reached even the basic conditions of survival, revealing the close link between environmental injustices and social inequalities. In addition to direct exposure in the workplace, domestic and environmental routes increased the risk, such as washing contaminated clothes and the irregular disposal of industrial waste [21,22]. The absence of epidemiological records and a public database on the exposed population reinforces institutional invisibility. Many workers live in neighboring municipalities and remain without diagnosis or specialized follow-up.

The underreporting is alarming: the Notifiable Diseases Information System (SINAN) recorded only four cases of occupational mesothelioma in Minas Gerais between 2004 and 2018 [10], and about 33% of deaths are not identified in official systems [16]. In direct contrast to underreporting, ABREA/MG recorded twelve cases in men in the recent period (2017 to 2022), with no corresponding investigation in women [21,61]. Studies in Italy indicate that 10.2% of cases have a non-occupational origin [62], reinforcing the urgency of expanded surveillance strategies, which has been carried out by civil society entities such as ABREA/MG.

The analysis of mesothelioma mortality in the Brazilian scenario, between 2009 and 2020, demonstrates a significant level of deaths and reflects the long latency period of the disease. In total, 739 deaths were recorded in men and 481 in women due to mesothelioma as the exclusive cause, resulting in a crude rate of 0.06 per 100,000 inhabitants for men and 0.04 for women (world standard rate of 0.06 and 0.03, respectively). The data reveal a significant concentration of deaths and a gradual increase in specific rates in the older age groups. From age 60 onwards, mortality rates skyrocket, peaking in the 80 years and older age group, where men recorded the highest rate (0.63) and women (0.32), highlighting the concentration of proportional deaths in the later age groups.

When analyzing the regional scenario, the mortality data for mesothelioma in the Belo Horizonte Health Region, where the exposure center is located, reinforce the disparity in reporting, as shown in Table 1.

This analysis of Table 1 is extremely concerning. The municipality of Confins has a crude mortality rate for mesothelioma in men of 2.65/100,000, a very high level when compared to the world standardized rate (0.06/100,000) [62]. However, the mortality rate for mesothelioma in women in the same region is zero (0.00) in all municipalities. This lack of records suggests that historical underreporting and the gender gap persist in the official accounting of asbestos-related illnesses.

The discrepancy is corroborated by the illness data recorded by ABREA/MG, which demonstrate a scenario of high incidence, especially among men, but with cases recorded in women that do not appear in the official mortality systems.

It is important to highlight that, throughout the exposure trajectory, women were systematically made invisible, and few broke through the barrier of seeking diagnosis. Nevertheless, the municipality of Pedro Leopoldo, characterized by high asbestos consumption, presented a mortality rate from ovarian cancer 34% above the national average (standardized rate ratio, SRR = 1.34) [63]. This data is especially worrying because, despite this excess, ovarian cancer is rarely institutionally associated with occupational or environmental exposure to asbestos, which reinforces the invisibility of women and the critical gaps in epidemiological surveillance.

The data analysis also reveals an important discrepancy in the investigation and recognition of diseases among women. The low proportion of female diagnoses observed in the records does not necessarily indicate less exposure, but points to persistent processes of underreporting, less clinical investigation, and less diagnostic prioritization of women, who were frequently exposed through domestic or occupational routes.

In summary, the results confirm the high incidence of asbestos-related diseases already recognized, while also revealing conditions not traditionally associated (non-classical illnesses), the occurrence of which demands new investigative approaches. These findings highlight gender inequalities in access to diagnosis and the essential role of organizations such as ABREA/MG, which play a fundamental role in making visible cases that would otherwise remain hidden.

It therefore reinforces the urgency of expanding health surveillance protocols, systematically incorporating a gender perspective, adopting more sensitive criteria to identify possible links between exposures and non-classical conditions, and strengthening the Unified Health System (SUS) network to guarantee the diagnosis and longitudinal follow-up of these workers and exposed individuals.

Access to specialized treatment, which requires travel to Belo Horizonte (approximately 80 km away), has deepened inequality in access to healthcare. In this context, ABREA/MG works in an articulated way between the victims and the SUS (Brazilian Public Health System), organizing transportation and solidarity support for consultations and exams, mitigating the gaps in state support.

The case of Pedro Leopoldo therefore synthesizes the multiple dimensions of environmental injustice: the exploitation of bodies and territories, gender inequality, and state omission. Even after the national ban on asbestos in 2017, contamination persists in the soil and in people’s lives. The territory remains a space of struggle for recognition, care, and historical reparation—a field in which science, ethics, and social justice need to act in an inseparable way.

## 4. Discussion

The evidence gathered indicates a systematic pattern of institutional silencing and omission, both on the part of the company and the state oversight bodies. This negligent and deceptive practice reflects a business conduct driven by the denial of risk and the manipulation of medical and environmental information [64]. It is noteworthy that the common business practice in the fiber cement sector is to conceal information about the risks of handling asbestos from workers and consumers [64,65].

[…everyone saw the error. Asbestos bags bursting, the machine breaking. And you couldn’t handle asbestos with your hand. So you would tear it open with your hand and throw it in there. Authorized by the mechanics manager, by the supervisor. They would order people to put on overalls and continue working that way. So it means: if you didn’t do it, you were fired. You were punished]

(H11)

The fraud was structural, based on intentional misinformation about the toxicity of chrysotile asbestos, the type used in the factory:

[…What they told us was that, in the beginning, chrysotile asbestos didn’t do any harm… But we know now that it was all to deceive us…]

(H34)

This abandonment and constant exposure led to the internalization of precariousness as something natural. The naturalization of risk, rooted in decades of exploitation and the absence of effective public policies, built a culture of silence and conformity in the face of collective illness. This dynamic, marked by misinformation and lack of institutional recognition, is also observed in other asbestos mining regions in Brazil [66]. The company’s medical service contributed to this silencing process by omitting diagnoses, avoiding official notifications, and restricting access to information about the real effects of exposure. The examinations were carried out in a way that concealed any mention of asbestos as a possible cause of the symptoms:

[“Your tests are good,” that’s all they ever said. Then every now and then they would come for me to get tested, but it’s no use getting tested. They used to call sometimes, now they don’t even call anymore. They fooled a lot of people there…”]

(H3)

The ineffectiveness of the inspection actions was guaranteed by the manipulation of labor inspections. According to the reports, the visits were announced in advance, allowing the company to simulate safety conditions:

[…Before the labor inspector came, most people had to stop to organize, to leave the factory very clean. They washed the whole factory, the clothes had to be clean, new clothes. They reduced the speed of the machine, of the equipment.] It would reduce by 90% of the percentage. Or else they would say that the factory was under maintenance so as not to create dust]

(H34)

Such practices reveal corporate collusion and regulatory ineffectiveness, constituting a violation of the fundamental principles of occupational health and safety provided for in Brazilian legislation [67,68,69]. This pattern of institutional fraud contributed to perpetuating the cycle of impunity, maintaining exposure levels far exceeding the limits recommended by the WHO (0.1 fiber/cm^3^), while the official Brazilian parameter was 2 fibers/cm^3^ [70].

This context of manipulation and silence resulted in significant human losses. Most research participants reported having lost a family member and/or close friend with suspected respiratory diseases and/or cancer, given that the diagnosis of ARD was avoided [21].

The testimonies reveal stories of illness and death of family members who were victims of direct and indirect exposure (occupational or environmental):

[…I dated this girl whose name was […] I married her after two years. After about two years of marriage, she developed malignant thyroid cancer. She underwent treatment, but it didn’t work, she passed away…]

(M5)

Another account adds:

[…My sister, unfortunately, died. She and her husband worked for many years at the factory. They both died young. My mother, before she died, confronted me: ‘Well, my dear, but I wasn’t contacted, my husband and I worked there.’ I kept quiet…]

(M28)

The collection of these accounts illustrates the circle of silence, invisibility and control [21] imposed on workers, in which the fear of dismissal, economic dependence and the absence of institutional support reinforced subservience and collective denial of the disease.

Faced with medical negligence, state and union omission, and the absence of reparation policies, a movement of collective resistance emerged among workers, led by ABREA/MG. This association, created by the workers themselves from 2014 and formalized in 2019, has become a central actor in the defense of health, recognition, assistance and the pursuit of socio-environmental justice.

ABREA/MG acts as a mediator between victims, research institutions and public bodies, articulating strategies for denunciation and sanitary, social and political support. The importance in the lives of those exposed was summarized in one of the meetings:

[…ABREA is breaking this prejudice within my family. There are already many associated members and they are continuing treatment. I see ABREA as a turning point in the asbestos issue, which came to make a difference, and I feel privileged and grateful to contribute a little…]

(CAP/ABREA/MG Meeting)

This mobilization resulted in public hearings and joint actions with the MPT/MG, increasing the visibility of the problem and pressing for the implementation of care policies for exposed populations. Two public collective actions for reparations for the damages caused are underway [61].

However, vulnerabilities and difficulties persist. The municipality has a limited structure for offering medium and high complexity services and lacks integrated surveillance. Thus, patients need to be referred to neighboring municipalities and, mainly, to Belo Horizonte, the state capital, traveling up to 80 km, especially in cases that require specialized diagnosis and more complex treatment [71,72]. Traveling to specialized care imposes logistical and financial barriers. A worker reported the difficulty of access and dependence on the association:

[…These pleural plaques that were diagnosed, I have to go back to the doctor, right? ABREA is the one that schedules them for me. I don’t go alone. One reason is that I don’t know how to get there, another is that it’s very bad, BH itself. It’s complicated for us…]

(H13)

This testimony shows that, for many, access to more complex health care is often made possible only thanks to the mediation and support of the association. Collective resistance is also expressed as a way of coping with suffering. By sharing experiences and claiming rights, ABREA/MG members transform pain into political action and silence into active memory. This praxis, rooted in the tradition of Latin American Collective Health and action research in creating expanded research communities [30,35,36], reinforces the ethical and emancipatory dimension of the struggle for recognition, reparation and social justice in the region studied.

The results of this research are, to a large extent, consistent with recent literature on risk underestimation, inadequacy of traditional screening criteria, and persistence of illness even after the end of exposure. Markowitz [73] argues that populations exposed to asbestos require specific lung cancer screening strategies, as the risk is not adequately captured by usual parameters focused only on smoking, as may be customary. Brims et al. [74] reinforce this convergence by showing that widely used risk criteria may fail to identify exposed individuals and cancer cases, which is consistent with what we describe as (in)visibility and institutional gaps in identification/monitoring. Huang et al. [75] and van Zandwijk et al. [76] also corroborate by demonstrating that there is a measurable and persistent burden of lung cancer attributable to asbestos. At the same time, an under-recognition of asbestos-related lung cancer as an oncological problem, which converges with our emphasis on the prolonged effects and the inadequacy of institutional responses to the risk and illness.

Huh et al. [77] is convergent in the essentials (long latency, association with exposure characteristics) in South Korea. Porzio et al. [78] move in the same direction as our discussion on gender asymmetries by drawing attention to biases and gaps in the health of women exposed to asbestos, but does so from a different outcome (colorectal cancer). In turn, Metintas et al. [79] reinforces the importance of environmental exposure for lung cancer, which is compatible with the territorial emphasis of our study; The difference is that, being a biomedical/epidemiological approach, they tend to treat underreporting and invisibility indirectly, while our results point to these processes as the central (institutional and social) object of analysis. In short, there are authors who directly corroborate our main results [73,76,79]; and others who are compatible, but shift the focus (epidemiological plan/cases and outcome/agenda), without offering a truly opposing direction [77,78]. In this context, it is noted that asbestos causes not only mesothelioma, but also lung, larynx, and ovarian cancer, among others [80].

The case of Pedro Leopoldo thus synthesizes the multiple dimensions of environmental injustice: the exploitation of bodies and territories, gender inequality, and state omission. Even after the national ban on asbestos in 2017, contamination persists in the soil and in people’s lives. The territory remains a space of struggle for recognition, care, and historical reparation—a field in which science, ethics, and social justice must act inseparably.

## 5. Conclusions

The experience of Pedro Leopoldo highlights a silent epidemic caused by occupational, domestic, and environmental exposure to asbestos, marked by corporate negligence and state omission. Even after the ban on industrial activity, the impacts persist in bodies, homes, and the territory, revealing a pattern of lack of protection, in which abandonment and denial of care operate as forms of managing life and death.

The combined action of the company and the State produced a true economy of death, sustained by misinformation, underreporting, and the normalization of illness as a cost of productivity. This health and environmental liability expresses structural inequalities of class, gender, and race that have historically determined which lives were considered expendable.

Overcoming this scenario of injustice and violence requires the implementation of structured public policies, which include the official recognition of exposed populations, the adoption of integrated health and environmental surveillance with a gender perspective, the guarantee of historical reparation, and due institutional accountability. Additionally, it is imperative to strengthen the SUS network for the longitudinal monitoring of those exposed and to provide strategic support to workers’ organizations, such as ABREA/MG, whose work has been fundamental in breaking the silence and guaranteeing minimum care.

The experience of ABREA/MG demonstrates that, even in the face of institutional violence and state abandonment, collective subjects are able to rebuild paths of resistance, care, and justice, transforming individual suffering into political power and historical demands. Amid policies of abandonment and invisibility, the collective construction of knowledge among workers, researchers, and institutions emerges as a gesture of affirmation of life.

By transforming social suffering into the systematic production of information, empirical data, and collective demands, this praxis makes visible the concrete conditions of asbestos exposure experienced by workers and populations in the region. Even operating in an adverse context, in which both the company and the union positioned themselves in opposition to the demands of those exposed, the workers’ protagonism in creating their own institution dedicated to defending health and life constitutes a fundamental link in social resistance, popular vigilance, and the fight against impunity. This reaffirm environmental and social justice as an inseparable ethical-political horizon for the future of Pedro Leopoldo and other regions exposed to asbestos. However, there are still uninvestigated developments that highlight the environment, the intersectionality of gender in the participation of the resistance movement, exposure and invisibility, and specific struggles.

## Figures and Tables

**Table 1:** Crude mortality rates for mesothelioma, per 100,000 inhabitants, by sex, in municipalities of the Belo Horizonte (MG) health region, between 2009 and 2020.

Municipalities	Men: Gross Rate (per 100,000)	Women: Gross Rate (per 100,000)
**Confins**	**2.65**	0.00
**Lagoa Santa**	0.00	0.00
**Matozinhos**	0.00	0.00
**Pedro Leopoldo**	0.55	0.00
**São José da Lapa**	0.77	0.00
**Vespasiano**	0.00	0.00

Source: Tables adapted from MS/SVS/DASIS/CGIAE/Mortality Information System–SIM MP/Brazilian Institute of Geography and Statistics Foundation–IBGE MS/INCA/Conprev/Surveillance Division.

## Data Availability

The original contributions presented in this study are included in the article. Further inquiries can be directed to the corresponding author.

## Abbreviations

The following abbreviations are used in this manuscript:

SUS	Unified Health System
ERC	Extended Research Community
PPE	Personal Protective Equipment
ARD	Asbestos-Related Diseases
SINAN	Information System for Notifiable Diseases
ABREA/MG	Brazilian Association of Those Exposed to Asbestos of the State of Minas Gerais
WHO	World Health Organization
MPT	Public Prosecutor’s Office for Labor
